# Appendiceal goblet cell adenocarcinoma newly classified by WHO 5th edition: a case report (a secondary publication)

**DOI:** 10.1186/s40792-024-01967-x

**Published:** 2024-07-09

**Authors:** Takayasu Azuma, Yoshihiro Sato, Hiroto Chiba, Junichiro Haga

**Affiliations:** 1 Department of Surgery, Yonezawa City Hospital, 6-36 Aioi-chou, Yonezawa, Yamagata 992-8502 Japan; 2 Department of Hepato-Biliary-Pancreatic and Transplant Surgery, Fukushima Medical University, 1 Hikarigaoka, Fukushima, Fukushima 990-1295 Japan

**Keywords:** Appendiceal goblet cell adenocarcinoma, Goblet cell carcinoid, Appendiceal tumor

## Abstract

**Background:**

Appendiceal goblet cell adenocarcinoma (AGCA) is a newly proposed cancer type in the 5th edition of the WHO Classification of Tumours in 2019. We experienced this rare form of appendiceal primary neoplasm.

**Case presentation:**

An 85-year-old male presented a positive fecal occult blood test. A series of imagings revealed a type 1 tumor, located on the appendiceal orifice. The subsequent biopsy made the diagnosis of signet-ring cell carcinoma. Consequently, he underwent the laparoscopic-assisted ileocecal resection. Initially, the tumor was suspected to be a Goblet cell carcinoid (GCC). There was a discrepancy between the histological and immunostaining findings: the tumor cells exhibited morphological similarities to GCCs, however displayed limited staining upon immunostaining. Ultimately, we concluded that the tumor should be classified as AGCA, by following WHO 5th Edition. AGCA represents a newly categorized subtype of adenocarcinomas. Because of our preoperative suspicion of malignancy, we performed tumor resection with regional lymph node dissection, despite the fact that most appendiceal malignant tumors are typically identified after an appendectomy.

**Conclusion:**

We experienced a case that provides valuable insights into the comprehension of AGCA, a recently established pathological entity in the WHO 5th Edition. This article is an acceptable secondary publication of a case report that appeared in Azuma et al. (J Jpn Surg Assoc 83:1103–1108, 2022)

## Background

Appendiceal endocrine neoplasms represent an infrequent subtype of primary tumors arising in the appendix. Among these, goblet cell carcinoid (GCC) stands out, exhibiting pathological characteristics that overlap with both carcinoid and adenocarcinoma. GCC has been previously categorized as a subtype of mixed adeno-neuroendocrine carcinoma (MANEC). However, its classification as a neuroendocrine tumor has been subject to considerable scrutiny due to the uncertain tumor origin, the use of the term “carcinoid” despite its high biological grade, and the varying nomenclature beyond GCC. In the year 2019, the 5th edition of the WHO Classification of Tumours was published, introducing the novel concept of appendiceal goblet cell adenocarcinoma (AGCA) [1]. This newly proposed classification emphasizes the adenocarcinomatous nature of GCC and stipulates its treatment as an adenocarcinoma. In light of these developments, initially suspected to be GCC but aligns more accurately with the newly defined AGCA. This case report was published at the Journal of Japan Surgical Association, 83: 1103–1108, 2022 [2]. This manuscript was submitted in an acceptable secondary publication with the approval of the editorial board of the *Journal of the Japan Surgical Association*.

## Case presentation

**Fig. 1:**
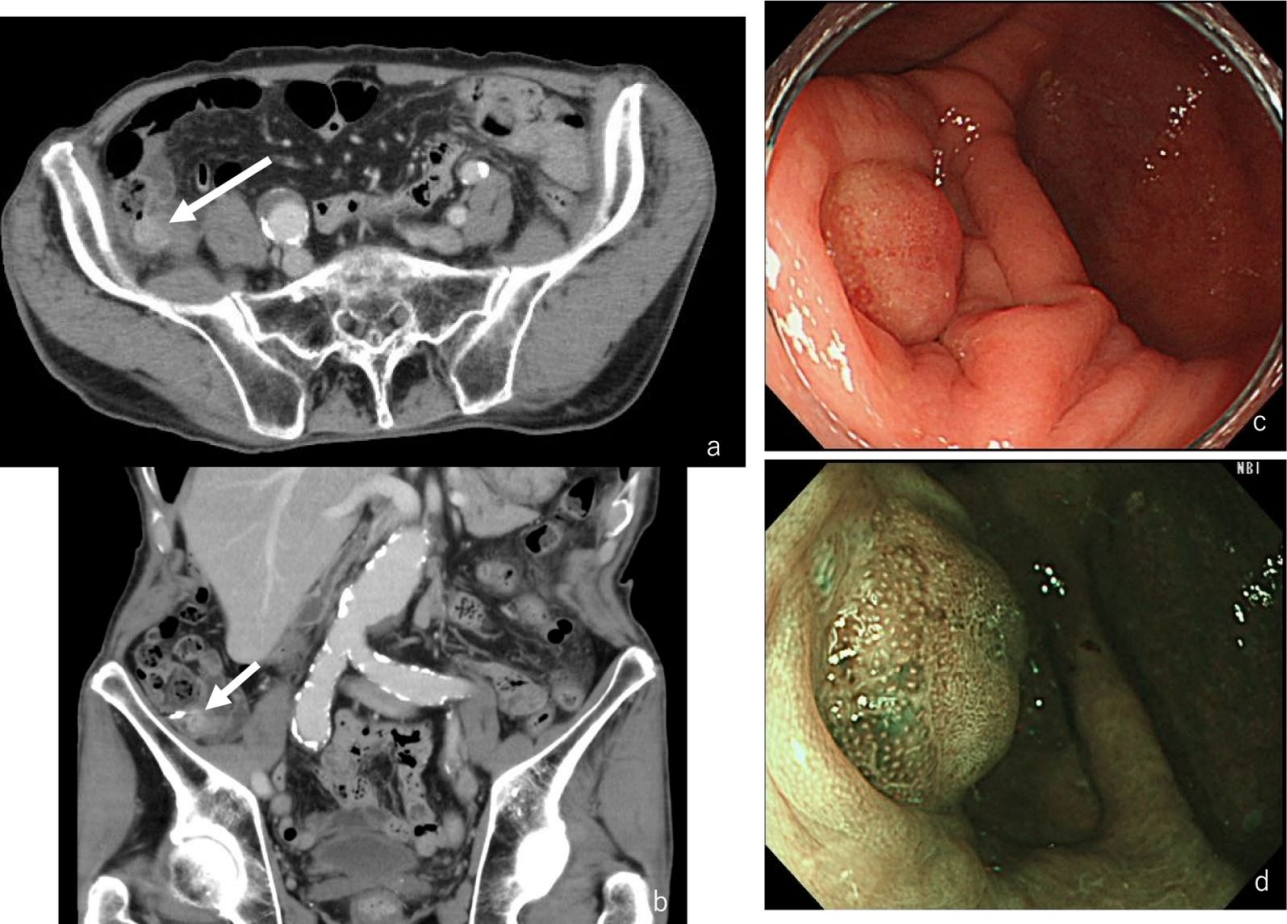
Image findings. **a**, **b** Contrast computed tomography scan findings. CT scan revealed a 15-mm nodular lesion with contrast effect at the appendiceal orifice. There was no metastatic lymph node swelling. **c**, **d** Colonoscopy findings. Colonoscopy showed a 10-mm elevated lesion at the appendiceal orifice

**Fig. 2:**
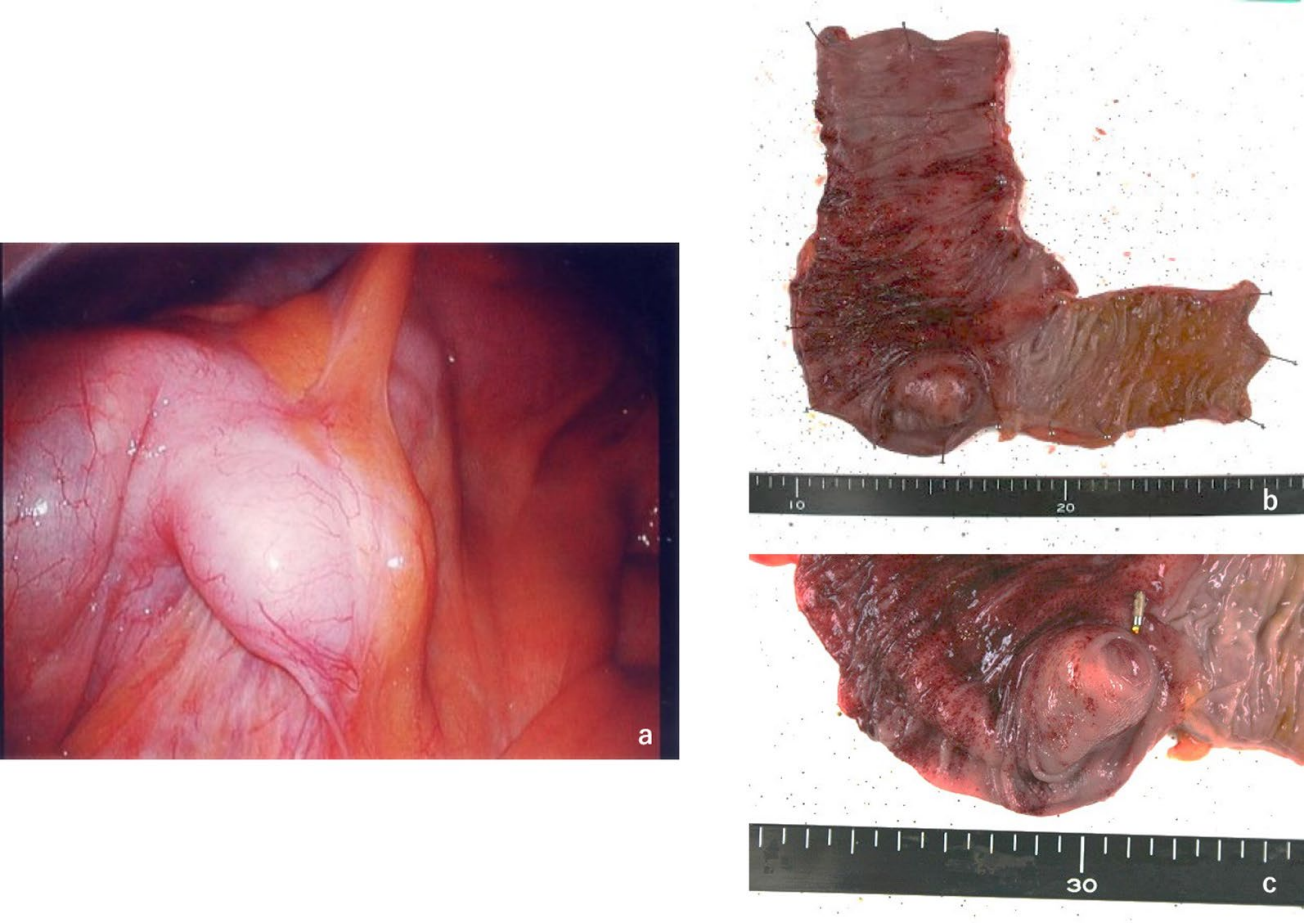
Operative findings. **a** Intraoperative findings. The tumor was not exposed on the serosal surface of cecum. No malignant enlarged lymph nodes were observed. **b** Surgical specimen. **c** The tumor was located at the appendiceal orifice

An 85-year-old male came to our hospital with a positive fecal occult blood test that was performed as part of colorectal cancer screening. Laboratory findings showed no abnormal values, including tumor markers. Abdominal computed tomography (CT) scan identified a 15 mm, enhanced nodular lesion on the appendiceal orifice (Fig. 1a, b). Any swelling lymph nodes suspected metastatic changes were not identified. The colonoscopy demonstrated a 10-mm elevated lesion on the appendiceal orifice (Fig. 1c, d). Endoscopic mucosal resection (EMR) was attempted, however it was unsuccessful because of the tumor size, thus only a biopsy was performed. The pathological finding was suspicion of signet-ring cell carcinoma. Consequently, we thought clinically signet-ring cell cancer of the cecum and performed laparoscopic-assisted ileocecal resection with regional lymph node dissection. Intraoperatively, it was revealed that the tumor was not exposed on the serosal surface of the cecum (Fig. 2 intraoperative findings). In the surgical specimen, there was a 15-mm elevated tumor known preoperatively on the appendiceal orifice, without abnormalities in the appendiceal mesentery (Fig. 2 surgical specimen).

**Fig. 3:**
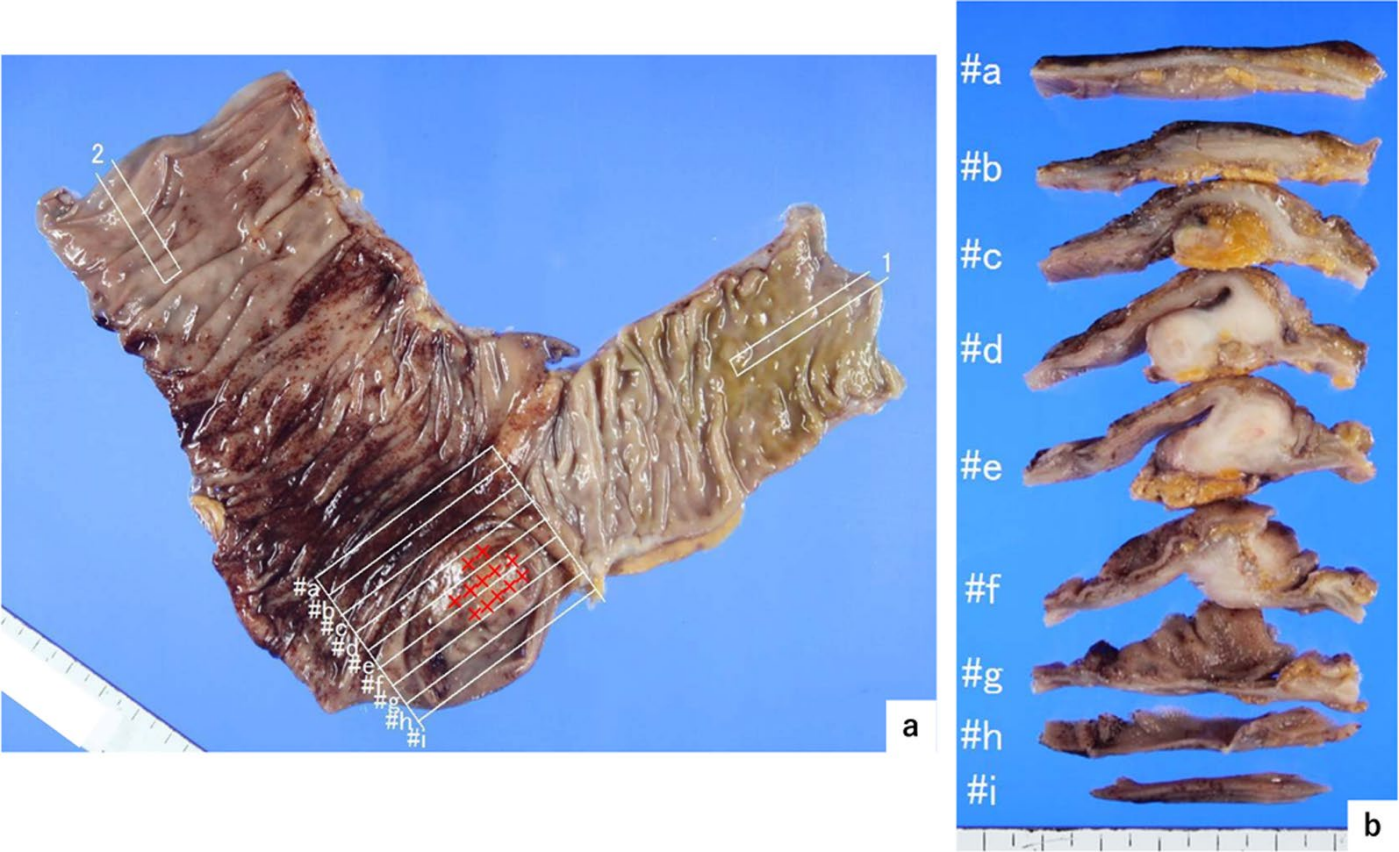
Histopathological findings. **a** Macroscopic findings. A smooth surface mass lesion was observed at the orifice of the appendix. **b** Tumor section

**Fig. 4:**
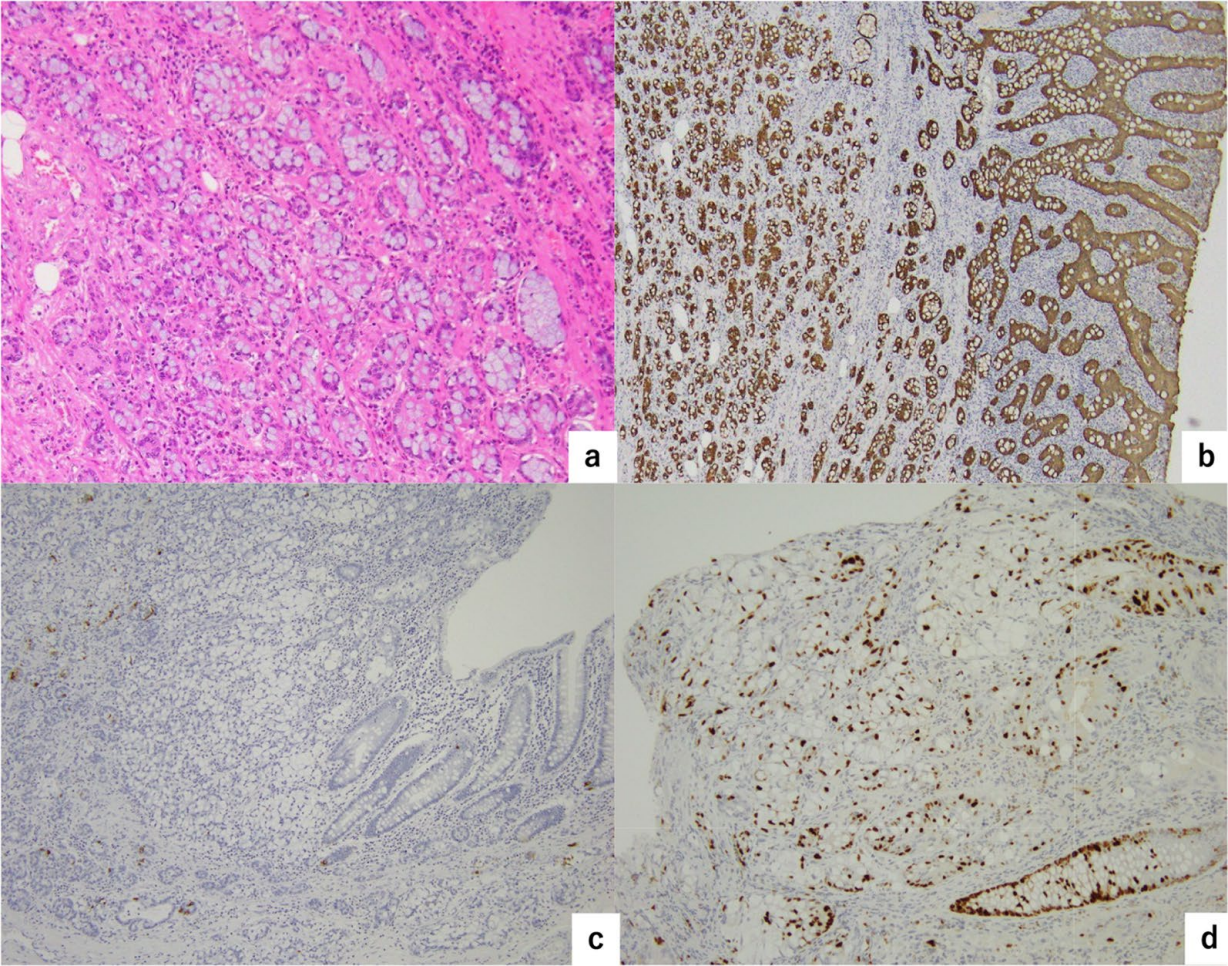
Immunohistochemical findings. **a** Hematoxylin and eosin staining × 40. Atypical cells tended to form clusters. **b** Cytokeratin staining × 40. The goblet cells grew in small clusters or solitary or trabecular pattern. **c** Chromogranin A staining × 20. The positive rate of chromogranin A was less than 1%. **d** MIB-1 (Ki67) staining × 20. The positive rate of MIB-1 was 45

Histopathological analysis of section #f from the tumor (Fig. 3b) showed a cluster of atypical cells reminiscent of goblet cells, characterized by mucus retention, detected within the mucosa, submucosa, and muscularis propria of the appendiceal structure. These cells displayed a distinctive growth pattern, forming small clusters, proliferating, and infiltrating in an isolated concentric circle arrangement. The atypical cells did not invade the serosa or mesentery of the appendix. And there was neither invasion into lymphatic and vascular vessels, however the tumor cells extended into the muscularis propria of the ileocecal region. The immunohistochemical analysis demonstrated that the atypical cells were negative for synaptophysin and CD56, with the chromogranin A positive rate being less than 1% (Fig. 4). Based on the pattern of atypical cell infiltration forming specific small clusters (Figs. 3, 4), we diagnosed Appendiceal GCC.

However, soon after our primary diagnosis, the new 5th edition of the WHO Classification of Tumours [1] was published, and we recognized that GCC was no longer included as a distinct entity. Instead, a new disease concept called appendiceal goblet cell adenocarcinoma (AGCA) was proposed. Following the 5th edition guidelines, we changed the diagnosis to AGCA.

AGCA is recommended to be graded based on the ability of atypical cells to form clusters (Table 1), and this case was diagnosed as Grade 2 because clustered atypical cell growth accounted for 50–75% of the total growth, despite a tendency for isolated growth in the mucosa-specific layer.

**Table 1:** Grading of appendiceal goblet cell adenocarcinoma [1]

Grade	Tubular or clustered growth (low-grade pattern) (%)	Loss of tubular or clustered growth (any combination of high grade)
1	> 75	< 25
2	50–75	25–50
3	< 50	> 50

Appendiceal goblet cell adenocarcinoma has been incorporated into the WHO 5th edition, and neuroendocrine characteristic are no longer obligatory for a definitive diagnosis. This case was diagnosed as Grade 2 because clustered atypical cell growth accounted for 50–75% of the total growth

The patient was discharged without complications, and adjuvant chemotherapy was not indicated due to his advanced age.

## Discussion

Primary appendiceal tumors are relatively uncommon, with reported incidence rates of primary appendiceal cancer ranging from 0.3 to 0.7% among all colorectal cancer resected surgically in Japan [3]. Appendiceal carcinoid constitutes 3.7% of primary appendiceal tumors and is even rarer among pancreatic and gastrointestinal neuroendocrine tumors (NENs), accounting for 7.4% in Japan [3]. Gagne [4] identified a distinctive type of appendiceal tumors that exhibited mucus-producing ability and possessed histopathologic characteristics of both carcinoid and adenocarcinoma, subsequently designated as Goblet cell carcinoid (GCC) by Subbuswamy [5]. However, the term “Carcinoid” is typically applied to pathologically very low-grade neuroendocrine tumors (NETs), whereas appendiceal GCC displays a relatively high biological grade and a low 5-year survival rate (73–83%) [6]. This discrepancy creates a dissonance between the nomenclature and the oncological features of the disease.

In the 4th edition of the WHO classification of tumors published in 2010, appendiceal Goblet cell carcinoid (GCC) was categorized as a subtype of mixed adeno-neuroendocrine carcinoma (MANEC), implying a higher grade compared to typical neuroendocrine tumors (NETs) in appendiceal endocrine neoplasms [7]. MANEC in the 4th edition of the WHO classification of tumors was also changed to mixed neuroendocrine-non-neuroendocrine neoplasm (MiNEN) in the 5th edition.

Pathological characteristics demonstrate that abnormal cells resembling goblet cells or signet-ring cells form trabecular, glandular, and foci-like structures; however, the majority form small aggregates, and some infiltrate and proliferate in isolation [7, 8]. In essence, the distinguishing feature of Goblet Cell Carcinoma (GCC) is the formation of small clusters by atypical cells, invading and proliferating, whereas signet-ring cell carcinoma always proliferates sporadically as solitary entities [1, 9, 10]. Additionally, some GCCs exhibit neuroendocrinological traits, and their immunostaining shows positivity for synaptophysin, chromogranin A, CD56, etc. [7]. Eeden et al. [11] reported that GCC is not always positive for neuroendocrine markers, but still 13% for synaptophysin, 44% for chromogranin A and about 6% for CD56.

In this case, the histopathological morphology demonstrated concordance with the appendiceal goblet cell carcinoma (GCC) histology as previously defined, albeit with little neuroendocrine expression (Fig. 4). Subsequently, in the 5th edition of the World Health Organization (WHO) classification published in 2019, the section pertaining to appendiceal GCC was omitted, and appendiceal goblet cell adenocarcinoma (AGCA) was introduced as a novel subtype of adenocarcinoma [1]. According to the WHO 5th edition, the histopathology of AGCA aligns with that of GCC in the 4th edition; however, it is explicitly stated that neuroendocrine characteristics are not obligatory [1].

The origin and development of tumor cells in appendiceal GCC still remain ambiguous. Furthermore, the existence of various diagnostic terms, including goblet cell type adenocarcinoid, mucinous carcinoid tumor, microglandular carcinoma, and crypt cell carcinoma, has contributed to confusion [12]. In the 5th edition of the WHO classification [1], AGCA is recognized as a distinct pathological entity separate from NETs and conventional adenocarcinomas. The TNM staging for AGCA is recommended to be conducted in accordance with appendiceal adenocarcinoma.

In the majority of cases, primary appendiceal tumors, including NETs, come to light post-appendectomy for acute appendicitis [6, 8]. Subsequently, based on the findings from the postoperative pathological examination, additional resection is frequently undertaken. However, in this particular case, we identified an appendiceal tumor through fecal occult blood testing. The preoperative diagnosis led us to suspect signet-ring cell carcinoma, enabling us to conduct a comprehensive operation with regional lymph node dissection in a single surgical procedure. Despite facing challenges in comprehending the histomorphological and immunostaining results, the introduction of AGCA in the 5th edition of the WHO classification provided an effective resolution in understanding this case.

We searched the PubMed database using the key words “appendiceal goblet cell adenocarcinoma”, between 2019 and 2023. 16 cases have been described in 11 reports, including ours (Table 2) [13–21]. Except for our case, most of the patients were relatively young, aged 53[47–59] years old. And in many cases, appendicectomy is performed first, and additional surgery is performed after the pathology results are received. In most cases, right-hemicolectomy has been chosen as the additional operation. Patients with lymph node metastasis or distant metastasis/peritoneal dissemination are treated with chemotherapy like that for colon cancer.

**Table 2:** Reported cases of appendiceal goblet cell adenocarcinoma between 2019 and 2023

Study	Patient	Initial operation	Additional surgery	TNM/stage	(Adjuvant)chemotherapy
Hennessy	38/F	–	Total colectomy with end ileostomy	TXNXM1c/IVC	Details unknown + HIPEC
	58/F	Lap Appe	Rt-HemiCole	T4aN1M1c/IVC	Details unknown
Kato	48/M	–	–	TXNXM1b/IVB	FOLFOX + panitumumab
	64/F	–	–	TXNXM1c/IVC	FOLFIRI
Sigley	57/M	Lap Appe	Rt-HemiCole	T3N0Mx/over IIA	NA
Lenti	52/F	Lap-assisted terminal ileum resection	Rt-HemiCole	T3N2Mx/IIIC	Details unknown
Nugent	53/M	Lap Appe	L-Rt-HemiCole	T4aN0M0/IIB	CapeOX(due to perforation)
Tamiya	63/M	Appe	Lap-ileocecal resection	T3N0M0/IIA → IVa(9 yr later)	FOLFOX + panitumumab
Goto	66/M	Lap Appe	Lap-ileocecal resection	T2N0M0/I	–
	46/F	Lap Appe	L-Rt-HemiCole	T3N0M0/IIA	–
	42/F	Lap Appe	–	T4aN0M1c/IVc	FOLFOX + bevacizumab
Kiyosawa	30/F	Appe	Lap-ileocecal resection	T3N0M0/IIA	–
	50/M	Lap Appe	Lap-ileocecal resection	T3N0M0/IIA	–
	60/M	–	Lap-caecal resection	T3N0M0/IIA	–
Koyama	54/M	Lap Appe	Lap-ileocecal resection	T4aN0M0/IIB	5-FU
Our case	85/M	Lap-assisted ileocecal resection		T3N0M0/IIA	–

*Lap* laparoscopic, *Appe* appendectomy, *Rt-HemiCole* right-hemicolectomy, *NA* not available, *HIPEC* hyperthermic intraperitoneal chemotherapy

Because AGCA is a rare disease, guidelines for its treatment have not yet been established. When AGCA is diagnosed after emergency appendicectomy for appendicitis, it remains unclear whether additional resection should be performed and, if so, which surgical technique should be chosen. In the National Comprehensive Cancer Network (NCCN) guidelines recommend right hemicolectomy and dissection of at least 12 lymph nodes for an accurate diagnosis in tumors with features such as lymphovascular invasion or positive margins [22]. Regarding chemotherapy, the NCCN guidelines recommend that AGCA be treated according to the treatment of colon cancer [22]. The prognosis of AGCA depends on the stage and grade of the tumor. Most low-grade AGCA are included in Stage I or II, but about 1/3 will metastasize. On the other hand, 50–70% of cases of high-grade goblet cell adenocarcinoma are included in Stage IV [1]. The most common sites of AGCA metastasis are the peritoneum, the omentum, the abdominal wall, and the ovaries. The overall survival for low-grade AGCA is 84–204 months, and 60–86 months for intermediates grade. The prognosis for high grade and disseminated cases is poor, ranging from 29–45 months [1]. The high biological grade of AGCA is evidenced by a case of recurrence 9 years later despite curative resection [18]. From this considerations, postoperative adjuvant therapy should be considered in cases with unfavorable character, as recommended by the NCCN.

## Conclusion

We present a case of appendiceal goblet cell adenocarcinoma, a recently defined entity in the 5th edition of the WHO classification. Due to its rarity and recent revision of the disease concept, there will be a need for considerable time to accumulate cases. Nevertheless, future consolidation of treatment methods will be necessary in the future.

## Data Availability

Not applicable.

## Abbreviations

AGCA: Appendiceal goblet cell adenocarcinoma
GCC: Goblet cell carcinoid
